# Comparison of 3D Hip Joint Kinematics in People with Asymptomatic Pronation of the Foot and Non-Pronation Controls

**DOI:** 10.21315/mjms2021.28.3.7

**Published:** 2021-06-30

**Authors:** Fayez Alahmri, Saad Alsaadi, Mohammed Ahsan

**Affiliations:** 1 Department of Medical Rehabilitation, Ministry of Health, Riyadh, Saudi Arabia; 2 Department of Physical Therapy, College of Applied Medical Sciences, Imam Abdulrahman Bin Faisal University, Dammam, Saudi Arabia

**Keywords:** pes planus, flat foot, kinematics, hip joint, gait, movement

## Abstract

**Background:**

The impact of asymptomatic pronation on proximal joints during motion has not been well understood, and research on it remains limited. Therefore, the current study determines the effect of asymptomatic pronation of the foot on hip joint kinematics during gait.

**Methods:**

Forty participants were recruited for the study (20 with asymptomatic pronated feet and 20 with non-pronated feet). Foot assessment was conducted by navicular drop and rear-foot angle tests. Hip joint kinematics were measured via MVN Xsens system 3D-motion capture from sagittal, frontal and transverse planes during gait. An independent *t*-test was used to identify differences in kinematic variables.

**Results:**

Both groups were similar in characteristics, and there were no significant differences between the groups in age (*P* = 0.674) and BMI (*P* = 0.459). However, there was a significant difference in arch height (*P* = 0.001) and rear-foot angle (*P* = 0.001). Our findings showed there were insignificant differences between the asymptomatic pronated foot and non-pronated foot control groups in hip joint kinematics of sagittal (*P* = 0.618), frontal (*P* = 0.276), and transverse (*P* = 0.337) planes during a full gait cycle.

**Conclusion:**

Patients with asymptomatic pronation of the foot and non-pronation of the foot showed similar movement patterns of hip kinematics in all three planes. The findings of the present study highlight the need for clinicians to consider foot alignment when examining patients with asymptomatic pronation of the foot and non-pronation of the foot.

## Introduction

Asymptomatic pronation foot is a chronic condition characterised by a complete or partial loss of the medial longitudinal arch (MLA) and associated with increased rear-foot eversion leading the foot to remain in a position of maximum pronation (1). The most important biomechanical function of bipedal gait is maintaining balance, shock absorption and body weight support (2). However, in people with pronated feet and due to the flattened arch, the foot cannot properly distribute the load from the bodyweight, which leads to biomechanical changes (3). These changes can be responsible for several musculoskeletal injuries of the lower limb, such as knee and lower back pain, Achilles tendinosis and stress fractures (4). In normal gait biomechanics, after initial contact with a surface, the subtalar joint begins to pronate until the head of the metatarsal contacts the surface and then the subtalar bone begins to supinate, which allows the foot to change from a flexible structure to a rigid structure, which helps the foot adapt to the ground more easily. However, for an individual with pronated feet, the foot remains in a pronated position without returning to supination (5, 6).

Foot pronation is often associated with internal rotation of the lower limb, whereas supination is associated with external rotation (7). Therefore, it is important to understand the motion transferred from distal to proximal and proximal to distal of a person with pronated feet. Distal injury is probably related to abnormal hip functions, while proximal injury is probably associated with abnormal foot structure (8). Foot pronation is responsible for pelvic malalignment. Tateuchi et al. (9) reported that a pronated foot leads to hip and pelvic malalignment with increased hip medial rotation in a unilateral standing position.

Furthermore, Khamis and Yizhar (10) stated that the pronated foot leads the pelvis to tilt anteriorly in standing position. Resende et al. (11) studied the impacts of a unilateral pronated foot on the pelvis and lower extremity biomechanics during gait by using wedged sandal to simulate foot pronation. They found an increase in adduction moments of the knee and hip, and also increased lower extremity internal rotation. There are, however, some limitations in the studies mentioned above. These studies recruited healthy participants with normal feet and used a platform to simulate a pronated foot. Subjects with a pronated foot have different muscle activity (12) and have different muscle strength (13), which would influence joint angles and kinematics.

The relationship between foot structure and lower limb alignment remains controversial (14). It has been reported that a pronated foot caused no significant difference in lower limb alignment abnormalities after a study was conducted on the effect of a pronated foot on lower limb alignment using three-dimensional radiograph reconstructions and comparisons with a normal arched foot (15). Moreover, a previous study found no kinematic differences in children with flat feet during gait, after a comparative study between children with flat feet and normal arched feet compared the three-dimensional kinematics of the lower limb joints (6).

The effects of asymptomatic pronation of the foot on hip joint kinematics during gait have not been well understood. Therefore, the current study investigates the effect of asymptomatic pronation of the foot on hip joint kinematics during gait.

## Methods

### Study Design and Setting

The study design was a cross-sectional design. The study took place in the biomechanics laboratory of Imam Abdulrahman Bin Faisal University in Dammam, Saudi Arabia in 2018.

### Sample Size Calculation

The sample size was calculated using power analysis, based on a study by Tateuchi et al. (9) that used internal/external hip rotation values of subjects with pronated feet. Mean hip rotation value of the control group (mu0 value) was 2.15°. Mean hip rotation value of the experimental group (mu1 value) was 9.52°, standard deviation (sigma) 7.8. This was based on a two-sided test alpha level of 0.01 and power value of 0.95. The sample consisted of 20 participants per group. Sample size analysis was conducted using the Department of Statistics at the University of British Colombia’s webpage: https://www.stat.ubc.ca/~rollin/stats/ssize/n1a.html

### Participants

Forty adult males voluntarily participated in the study. Twenty had asymptomatic pronated feet and 20 with non-pronated feet were used as a control group. Both groups were recruited from Imam Abdulrahman Bin Faisal University through advertisements. Participants were initially screened using inclusion and exclusion criteria.

## Inclusion Criteria

Subjects in the experimental group were included in the study if their arch height changed by more than 10 mm as measured by the navicular drop (ND) test and if their rear-foot (calcaneal) angle measured larger than 5°. The control group was selected based on the following criteria: normal foot arch with an arch height change of less than 10 mm in the ND test.

## Exclusion Criteria

Subjects were excluded if they had any foot deformities, systemic or neurological diseases, or a history of foot or ankle surgery or a recent fracture.

## Navicular Drop Test

ND was measured to assess MLA arch height; ND was calculated by measuring the difference between ND in open kinetic chain (non-weight bearing) and closed kinetic chain (with weight-bearing). The ND test has been proven to be a valid and reliable test (16). The amount of ND was measured in mm. A ND under 10 mm is considered normal and more than 10 mm as a flat arch (16).

## Rear-Foot Angle

Rear-foot angle measurement is a clinical method that has been accepted in many studies (17–19). The measurement assesses calcaneal eversion or heel valgus and determines the amount of foot pronation. Kanatli et al. (20) stated that rear-foot angle and MLA foot arch height must be considered separately in flat foot assessment. The rear-foot angle was measured with the subject lying in the prone position and the examined foot and ankle extended 10 cm out of bed. A longitudinal line was drawn with a pen along the posterior aspect of the lower third of the leg and then the subject stood on both feet. The rear-foot angle was measured using a goniometer as the angle between the calcaneus and the lower third of the leg.

## Hip Joint Kinematics

Gait kinematics were measured by the MVN Xsens system (MVN, Xsens Technologies BV, Netherlands), a 3D motion capture inertial-based sensor used to capture and analyse the hip kinematics. The recorded motion was visualised and processed with MVN studio software. MVN Xsens consists of a full-body suit equipped with sensors placed at the lower and upper limbs. The MVN Xsens system has exhibited moderate to high reliability and validity in assessing human kinematics (21, 22).

### Lab and Subject Preparation

The system was calibrated according to the manufacturer guidelines and data was transmitted to a laptop over WiFi using company software (MVN Studio version 2018). The steps of the measurements were explained to each subject. General information on the subjects, including height; width of shoulders and pelvis; and leg, knee and ankle length, was recorded. The subjects were asked to wear a full body suit. The sensors were placed at the shoulders, lower arms, upper arms, hands, lower legs, upper legs, feet, pelvis, sternum and head.

## Testing Protocol

Those who met the study eligibility criteria were asked to sign a consent form if they agreed to participate. Upon the participants’ arrival at the lab, the researcher measured their body weight and height and asked them to fill out a questionnaire. The dominant leg was recorded for each participant by asking him which leg they used to kick a ball. The recording session began with a calibration phase (‘N-pose’ calibration) to ensure the alignments between body segments and sensors were accurate so that estimates of joint positions and segment orientations could be made. During the calibration, the subject stood erect with his arms close to the body for a few seconds. Then, the testing began, with the subject walking barefoot at a natural speed. Five successful trials were captured and processed. Each trial involved one gait cycle beginning with a heel strike and ending with a prior heel strike of the same foot. Then, the data were analysed using MVN Studio software, version 2018.

## Statistical Analysis

All statistical analyses were performed using IBM SPSS software (version 20). Before analysis, the data were checked for normality and outliers. The data are presented as mean values and standard deviation. Independent *t*-tests were conducted between asymptomatic pronated feet and non-pronated feet participants for kinematic variables of the hip in sagittal (flexion/extension), frontal (abduction/adduction) and transverse (internal/external rotation) planes during gait. A *t-*test was also conducted for age, height, BMI, arch height and rear-foot angle. An α-level of 0.05 was used to determine statistical significance.

## Results

### Participant Characteristics

A total of 40 adult males participated and completed the study, 20 with asymptomatic pronated feet and 20 with non-pronated feet. Table 1 illustrates the participants’ characteristics.

There were no significant differences between both groups in terms of age (*P* = 0.674), height (*P* = 0.130), body weight (*P* = 0.278), and BMI (*P* = 0.459). There was a significant difference in the arch height (*P* = 0.001) and rear-foot angle (*P* = 0.001)

## The Effect of the Pronated Foot on Hip Joint Kinematics

There were no significant differences between both groups in hip joint kinematics during gait in all three planes: sagittal, frontal and transverse.

**Figure 1:**
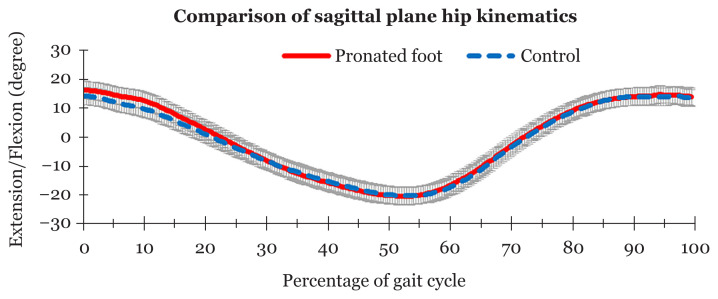
Comparison of means and standard deviations of sagittal plane hip kinematics between asymptomatic pronated feet and non-pronated feet

Table 2 shows that there were no significant differences in peak flexion (*P* = 0.298) and peak extension (*P* = 0.833) between the groups. The means of the hip kinematics in the sagittal plane (flexion and extension) did not differ significantly between the groups (*P* = 0.618). The asymptomatic pronated feet group and the non-pronated group showed a similar movement pattern (Figure 1).

**Figure 2:**
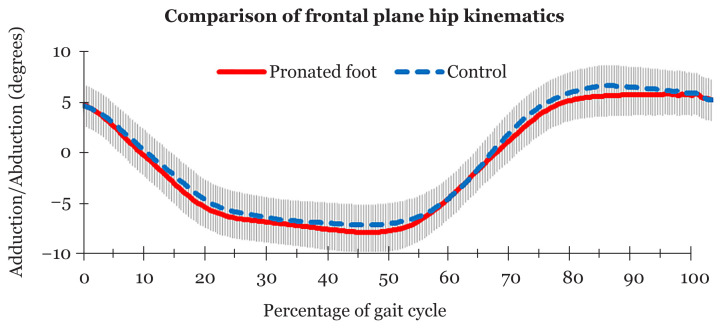
Comparison of means and standard deviations of frontal plane hip kinematics between asymptomatic pronated feet and non-pronated feet

Table 3 shows that there was no significant difference in peak abduction (*P* = 0.667) and peak adduction (*P* = 0.134) between the groups. The means of the hip kinematics in the frontal plane (abduction and adduction) also do not differ significantly between the groups (*P* = 0.276). The asymptomatic pronated feet and non-pronated feet groups showed a similar movement pattern (Figure 2).

**Figure 3:**
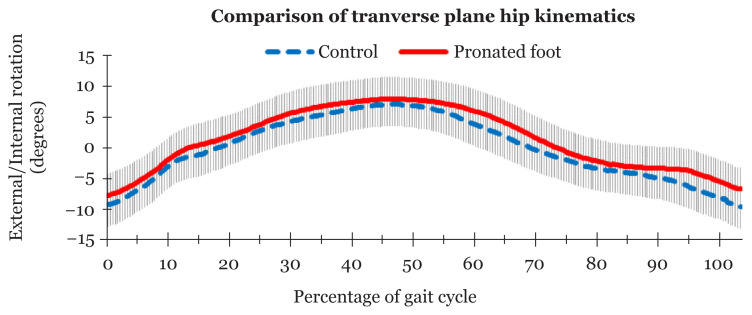
Comparison of means and standard deviations of transverse plane hip kinematics between asymptomatic pronated feet and non-pronated feet

Table 4 shows that there was no significant difference in peak internal rotation (*P* = 0.407) and peak external rotation (*P* = 0.118) between the groups. The means of the hip kinematics in the transverse plane (external/internal rotation) did not differ significantly between the groups (*P* = 0.337). The asymptomatic pronated feet and non-pronated feet groups showed a similar movement pattern (Figure 3).

## Discussion

This study investigated the effect of asymptomatic pronation of the foot on hip joint kinematics during gait. Our findings showed that there were no significant differences in the hip joint kinematics flexion/extension, abduction/adduction and internal/external rotations between the groups during the full gait cycle (stance to the swing phase). This result was the opposite of our hypothesis; our findings contradict previous studies that suggested that a pronated foot would alter lower limb kinematics (9, 10, 21, 23). This discrepancy may be due to the population investigated. The abovementioned studies recruited healthy participants with a normal foot arch and used a platform wedge or sandal to create foot pronation (medially tilted wedges used to simulate foot pronation). Regardless of the neuromuscular compensation that may compensate for any alteration in joint kinematics, subjects with a pronated foot may have different muscle activity and muscle strength, which would influence joints angles (12, 13). Therefore, it’s difficult to generalise their result to include people with pronated feet, as in our study.

A second potential reason for this discrepancy may be the nature of hip assessment. The current study investigates hip kinematics during motion. Joint alignment during motion is different from alignment in a static position; during movement, things are more complex because muscle forces are engaged and play a big role (5). Another limitation of the previous studies is the techniques they used for classifying and assessing foot posture. Our results showed that pronated feet and control groups shared similar hip joint movement patterns in sagittal, frontal, and transverse planes, which concurs with previous reports (6, 15). A similar conclusion was drawn by Cebulski-Delebarre, who found no significant difference in alignment abnormalities in the lower limb between a flat foot and a normal arched foot among individuals with a flat foot using 3D radiograph reconstructions of the lower extremities. The findings on adult subjects are similar to children with pronated feet. It has been reported that children with asymptomatic pronated feet are not significantly different from normal children during gait (24). It should be pointed out that flexible flat foot is not a major risk factor for other musculoskeletal lower limb injuries. Furthermore, Shih et al. (6) found no kinematic differences in children with flat feet during gait after a comparative study between children with flat feet and non-pronated feet; they compared the three-dimensional kinematics of the lower limb joints. They concluded that lower limb movement patterns were similar between the groups.

The literature has shown that flexible flat foot rarely causes disability (14, 25). A cohort study investigated the correlation between injuries and foot postures in a large number of participants, over 900 runners; they found that pronated foot is not related to an increase in the risk of injuries and there was no difference between pronated feet and non-pronated feet in regard to lower limb injuries. They concluded that they disagree with the common belief that a pronated foot increased risk of injury to the lower limb (14). Those with asymptomatic flexible pronated foot have developed an adaptive way to accommodate the structural deformity. It has been found that people with asymptomatic pronated foot demonstrated higher and different muscle activations, and that may be considered a part of a compensation mechanism to overcome the deformity (12).

This study had some limitations. The initial limitation was that the methods by which we measured the hip joint kinematics were possibly not sensitive enough to register a small difference. The current study did not investigate static alignment of the lower limb prior to the motion testing, which would illustrate if there were any abnormalities in a static position. The findings of this study can provide important information for the clinical assessment of individuals with asymptomatic pronated feet. The relationship between proximal joint injuries and asymptomatic pronated feet may not mainly be mediated by mechanical factors and other confounding factors related to both an asymptomatic pronated foot and proximal joint injuries. The results of the current study demonstrate a new perspective that asymptomatic pronated feet might not be responsible for lower limb injuries, in opposition to the predominant belief. Finally, this study proved that static malalignment does not necessarily affect motion.

## Conclusion

This study showed that subjects with asymptomatic pronation of the foot and nonpronation of the foot demonstrated a similar movement pattern of hip joint angles during gait in all three planes: sagittal (flexion/extension), frontal (abduction/adduction) and transverse (external/internal rotation).

## Figures and Tables

**Table 1:** Participant’s characteristic

Characteristics	Asymptomatic pronation foot (*n* = 20) mean (SD)	Non-pronation foot (*n* = 20) mean (SD)	*P*-value
Age (years old)	(4.12) 21.45	21.00 (2.54)	0.674
Height (cm)	(5.00) 173.35	169.73 (6.00)	0.130
Weight (kg)	73.00 (12.00)	68.00 (16.00)	0.278
BMI (kg/m2)	24.61 (4.12)	23.50 (4.76)	0.459
Arch height ND (mm)	13.53 (1.92)	7.42 (12)	0.001*
Rear-foot angel (degree)	8.21 (1.93)	3.52 (0.72)	0.001*

Notes: BMI = body mass index; SD = standard deviations;

* level of significant = *P* < 0.05

**Table 2:** Comparison of the hip kinematic parameters in the sagittal plane

Kinematic variables	df	Asymptomatic pronation foot (*n* = 20) mean (SD)	Non-pronation foot (*n* = 20) mean (SD)	*t*	*P*-value
Peak hip flexion (degrees)	38	17.44 (2.95)	16.61 (1.95)	1.06	0.298
Peak hip extension (degrees)	38	21.75 (3.06)	21.52 (3.78)	0.21	0.833

Note: Level of significant = *P* < 0.05

**Table 3:** Comparison of the hip kinematic parameters in the frontal plane

Kinematic variables	df	Asymptomatic pronation foot (*n* = 20) mean (SD)	Non-pronation foot (*n* = 20) mean (SD)	*t*	*P*-value
Peak hip abduction (degrees)	38	8.29 (2.57)	8.60 (1.77)	0.43	0.667
Peak hip adduction (degrees)	38	9.56 (2.27)	8.63 (1.48)	1.53	0.134

Note: Level of significant = *P* < 0.05

**Table 4:** Comparison of the hip kinematic parameters in the transverse plane

Kinematic variables	df	Asymptomatic pronation foot (*n* = 20) mean (SD)	Non-pronation foot (*n* = 20) mean (SD)	*t*	*P*-value
Peak hip internal rotation (degrees)	38	8.31 (2.86)	7.57 (2.77)	0.84	0.407
Peak hip external rotation (degrees)	38	8.98 (3.58)	10.95 (4.16)	1.59	0.118

Note: Level of significant = *P* < 0.05
